# Past, Present, and Future of Deep Brain Stimulation: Hardware, Software, Imaging, Physiology and Novel Approaches

**DOI:** 10.3389/fneur.2022.825178

**Published:** 2022-03-09

**Authors:** Jessica Frey, Jackson Cagle, Kara A. Johnson, Joshua K. Wong, Justin D. Hilliard, Christopher R. Butson, Michael S. Okun, Coralie de Hemptinne

**Affiliations:** ^1^Department of Neurology, Norman Fixel Institute for Neurological Diseases, University of Florida, Gainesville, FL, United States; ^2^Department of Neurosurgery, University of Florida, Gainesville, FL, United States

**Keywords:** deep brain stimulation, hardware advances, software advances, closed-loop, targeting strategies, connectomics, novel waveforms

## Abstract

Deep brain stimulation (DBS) has advanced treatment options for a variety of neurologic and neuropsychiatric conditions. As the technology for DBS continues to progress, treatment efficacy will continue to improve and disease indications will expand. Hardware advances such as longer-lasting batteries will reduce the frequency of battery replacement and segmented leads will facilitate improvements in the effectiveness of stimulation and have the potential to minimize stimulation side effects. Targeting advances such as specialized imaging sequences and “connectomics” will facilitate improved accuracy for lead positioning and trajectory planning. Software advances such as closed-loop stimulation and remote programming will enable DBS to be a more personalized and accessible technology. The future of DBS continues to be promising and holds the potential to further improve quality of life. In this review we will address the past, present and future of DBS.

## Introduction

Deep brain stimulation (DBS) has evolved substantially over the past several decades. The technology first appeared in mainstream practice in the 1980's for the treatment of Parkinson's disease (PD). Since then, innovative updates to DBS technology have led to an overwhelming expansion in its use and its application(s). Technical advances include more lead contacts and an increased number of algorithms and stimulation patterns as well as an emergence of increasing treatment indications. This narrative review will summarize the history of DBS development, conventional technology, and recent advances in DBS technology, including targeting strategies as well as hardware and software enhancements.

## History of DBS

The use of electrical stimulation to modulate brain activity dates back to ancient times, with electric fish being used to treat a range of neurological illnesses including headache and seizures (1). However, the development of modern DBS technology began in 1947, with the introduction of an innovative stereotactic apparatus by Spiegel and Wycis referred to as “stereoencephalotomy.” This tool was used for localization of ablative procedures (2). This new approach resulted in an improved mortality rate from 15 to 1%, which led to rapid growth of stereotactic neurosurgical procedures for a variety of neuropsychiatric disorders (3, 4). At this time, stimulation was predominantly being used to localize areas for selective brain ablation and as a method to avoid side effects (1). The use of intraoperative stimulation in patients with tremor led to the observation that lower frequency stimulation (5–10 Hz) exacerbated motor symptoms, whereas high-frequency stimulation (50–100 Hz) led to a reduction in symptoms (1, 3, 5, 6). In 1952, Jose Delgado began experiments with implanted electrodes in animals and humans, along with corresponding “stimoceivers” in the skull that could facilitate remote activation of the stimulation (1, 7). Around the same time, controversial psychiatrist Robert Heath developed 100-Hz chronic stimulation targeted at the septal region of the brain for the treatment of schizophrenia and pain (7). Neuroscientist Natalia Petrovna Bekthereva and neurophysiologist and psychiatrist Carl Wilhelm Sem-Jacobsen independently explored chronic neurostimulation as a means to create a lesion at whichever site yielded the best therapeutic results in conditions ranging from hyperkinetic disorders to epilepsy (1, 2, 7). Over the next two decades, PD and tremor became the main conditions treated with ablative stereotactic surgery, with over 25,000 surgeries completed in the PD patient population by 1968 (3).

Stimulation paradigms continued to be explored throughout the 1970's as a treatment for neurological disorders and for chronic pain, with advances occurring concomitant to substantial improvements in implantable medical devices, including spinal cord stimulators and cardiac pacemakers (1, 3, 8). Industry established divisions dedicated to the improvement of neurologic medical devices and in 1975, Medtronic Inc, was the first company to trademark the term “DBS” for deep brain stimulation (3). In 1980, DBS for the treatment of neurologic symptoms including dystonia, tremor, and speech impairment was first reported (9). This was followed up in the late 1980's by Benabid and colleagues, who reported successful chronic electrode implantation in the ventral intermediate (VIM) nucleus of the thalamus for treatment of tremor with DBS, in both essential tremor (ET) and PD (10). Following a series of studies which demonstrated that DBS induced fewer permanent side effects compared to lesional techniques, there was a movement toward DBS over ablative procedures especially when bilateral procedures were necessary (1). Enthusiasm for this technology increased in parallel with the development of tools that enabled objective assessment of the effects of DBS as well as a better understanding regarding disease pathophysiology. These developments included the Unified Parkinson's Disease Rating Scale (UPDRS), the identification of new therapeutic targets for DBS based on groundbreaking research involving basal ganglia circuitry, and the discovery of neurotoxin-induced non-human primate models of PD (3, 11).

DBS targeted to the ventral intermediate (VIM) nucleus of the thalamus for use in ET and severe PD tremor received a CE Mark and FDA approval in 1993 and 1997, respectively. Since then, indications for DBS have expanded to encompass a variety of movement disorders and neuropsychiatric indications, targeting brain structures such as the subthalamic nucleus (STN), the globus pallidus internus (GPi), and the original thalamic target in the VIM. Currently, DBS has obtained a CE Mark and FDA approval for ET (VIM), PD (VIM, STN and GPi), and epilepsy (anterior nucleus of the thalamus; ANT), and a humanitarian device exemption for dystonia (STN and GPi) and obsessive-compulsive disorder (anterior limb of the internal capsule; ALIC) (12). DBS is currently being investigated as a potential treatment for Tourette Syndrome with promising initial results (13) and for major depression and Alzheimer's disease (14), although results have been limited in numbers (15). Finally, a variety of case reports and small case series have described experimental uses of DBS for indications including anorexia, obesity, addiction, and chronic pain, among others (16).

## Overview of DBS Technology: Conventional Hardware and Advances

The basic components of DBS include the internal system, consisting of the lead and electrodes, the extension cables, and the implantable pulse generator (IPG), as well as the external system, consisting of the clinician programmer, the patient programmer, and a recharger for rechargeable devices (12). The lead is composed of an electrode array, variable in length, which is inserted stereotactically into a specific brain target. The lead is then attached via extension cables to the IPG, which is typically located in the anterior chest or abdomen, depending on individual patient anatomy and preference.

The technology that has been developed for DBS is largely dependent on what has been produced by the various DBS system manufacturers. The three original DBS system manufacturers are Medtronic, Abbott (formerly St. Jude Medical), and Boston Scientific. More recently, PINS Medical and SceneRay are two DBS system manufacturers from China, and Newronika is a company from Italy that have developed alternative DBS systems. Each of these companies continues to make advancements to DBS technology, yielding more innovative software and hardware to improve therapeutic outcomes for patients.

### Electrodes

**Figure 1:**
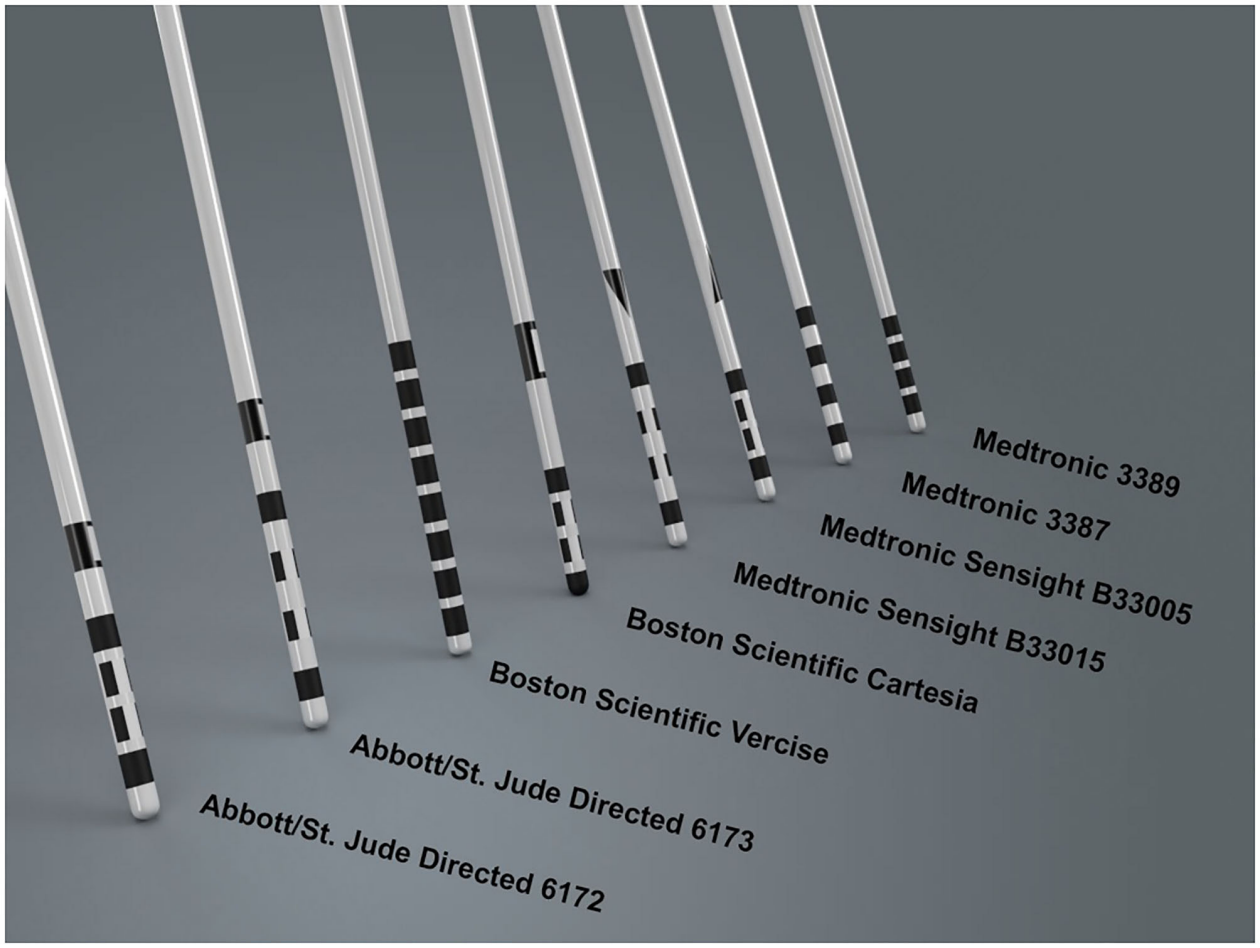
Lead design currently commercially available from various DBS manufacturers. Contacts are either full rings, allowing for omnidirectional stimulation, or have segmented electrodes on the middle two levels, allowing for directional stimulation. Many manufacturers include stereotactic markers above the DBS contacts for post-operative directional lead orientation.

The materials used to construct the electrodes are important to consider. Currently, commercially available DBS electrodes are composed of platinum-iridium with nickel alloy connectors encased in a polyurethane sheath (7). Platinum-iridium is inert, maintains good electrical properties with continuous stimulation, and has low impedance, making it a favorable material for use in brain tissue (17). In addition, the iridium component adds a useful and practical stiffness to the electrode (17). Conventional leads are composed of 4 electrode ring contacts that are 1.5 mm in length. These contacts are spaced either 0.5 or 1.5 mm apart on a cylindrical electrode that is 1.27–1.36 mm in diameter (18). Commercially available leads vary and are selected based on the brain area being targeted and the therapeutic indication (Table 1, Figure 1).

**Table 1:** Description of currently available leads and their basic parameters including number of contacts and sizes of contacts.

**Manufacturer**	**Lead Type**	**Number of Contacts**	**Lead Diameter (mm)**	**Length of Contacts (mm)**	**Spacing Between Contacts (mm)**
Medtronic	3387	4	1.27	1.5	1.5
	3389	4	1.27	1.5	0.5
	3391	4	1.27	3.0	4.0
	Sensight BM33005	8 (1-3-3-1)	1.36	1.5	0.5
	Sensight BM33015	8 (1-3-3-1)	1.36	1.5	1.5
Abbott	6166 and 6168	4	1.29	1.5	0.5
	6167 and 6169	4	1.29	1.5	1.5
	6170 and 6172	8 (1-3-3-1)	1.29	1.5	0.5
	6171 and 6173	8 (1-3-3-1)	1.29	1.5	1.5
Boston Scientific	Linear 8-contact lead (DB-2201-30DC/DB-2201045DC)	8	1.3	1.5	0.5
	Cartesia directional lead (DB-2202-30/DB-2202-45)	8 (1-3-3-1)	1.3	1.5	0.5
PINS medical	L301 and L301S	4	1.3	1.5	0.5
	L302 and L302S	4	1.3	1.5	1.5
SceneRay	1200	4	1.27	1.5	0.5
	1210	4	1.27	1.5	1.5

**Figure 2:**
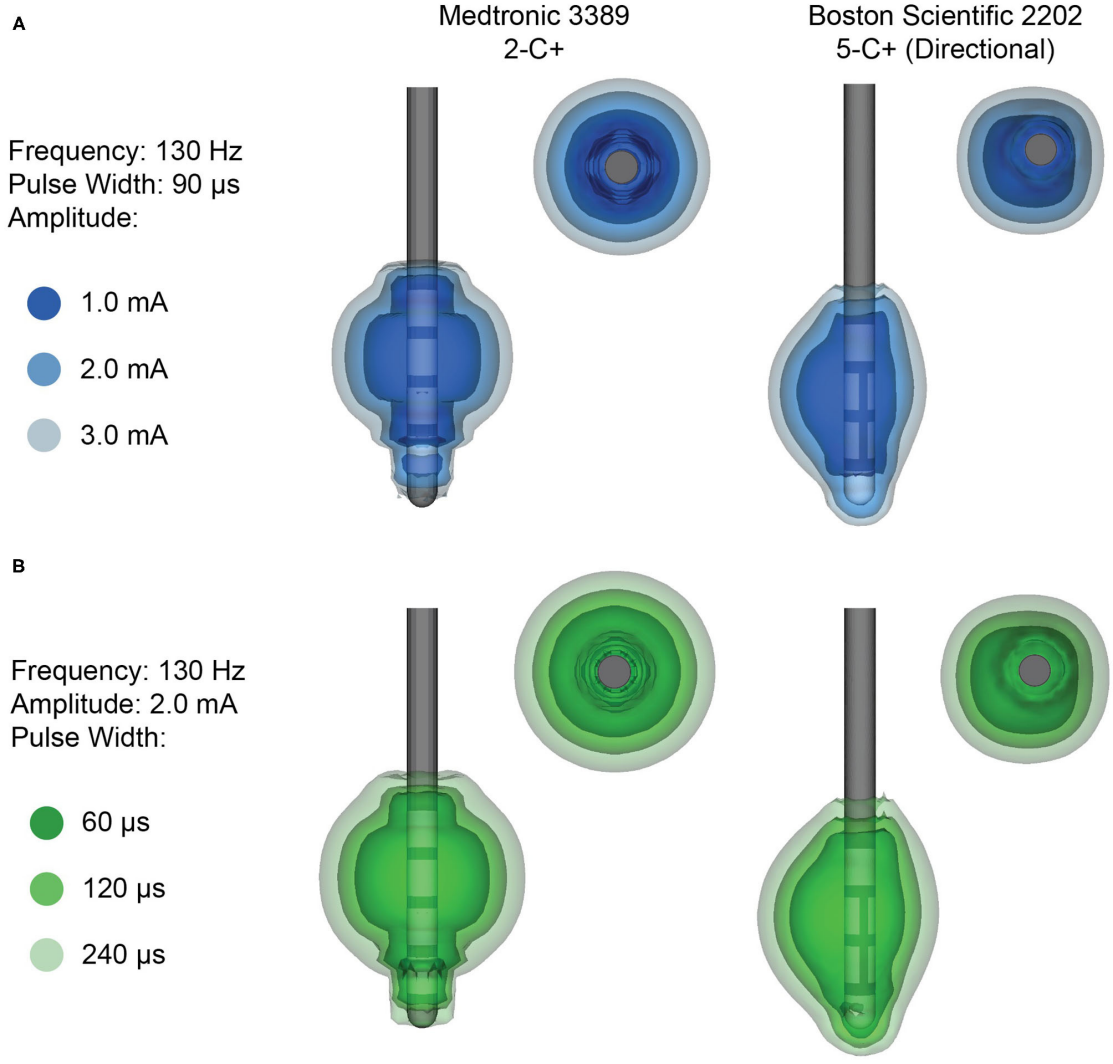
Example volumes of tissue activated (VTA) for clinical stimulation parameters. **(A)** Increasing amplitude and **(B)** increasing pulse width results in a larger VTA. VTA are shown for monopolar stimulation from the Medtronic 3389 lead (left), which delivers omnidirectional stimulation, and the Boston Scientific 2202 lead (right), which is capable of steering the stimulation with directional contacts.

The electrode design plays a crucial role in the stimulation capabilities of the DBS system and the cylindrical ring-electrode design is limiting for a variety of reasons. The volume of tissue activated (VTA) is a modeling technique used to estimate the brain tissue that may receive stimulation (19). The VTA allows for a gross representation of the brain areas that could potentially be stimulated. In reality, the neurons that are ultimately stimulated depends on several factors, including distance from the cathode, fiber type (i.e., myelinated vs. unmyelinated), and fiber orientation (19). Although the exact anatomical area that is stimulated cannot be precisely determined, the VTA can be used to estimate the projected electric field and the corresponding behavior of adjacent brain tissue in response to the electric field gradient (20). The VTA is dependent not only on which contact is used for stimulation, but also the total number of contacts used and their polarities, the stimulation parameters chosen (including pulse width, current orientation and amplitude, and frequency), and the properties of the surrounding tissue (Figure 2). In a conventional electrode design, the VTA is shaped along the z-axis of the lead, typically resulting in a symmetric, omnidirectional VTA (7, 15). However, it is important to point out that VTA models typically use a isotropic conductivity (that is, electric conductivity is equivalent in every direction) tissue model, whereas the biophysical properties of brain tissue are anisotropic and would lead to asymmetric tissue conductivities and electric field gradients (19, 21). There have been some attempts to better and more accurately characterize the VTA using heterogenous biophysical tissue models, although this is an area that requires further exploration (21).

The therapeutic area is bordered by a variety of structures that could induce side effects when stimulated, such as the internal capsule surrounding the STN. A theoretical VTA may be helpful for determining whether the therapeutic area can be covered while minimizing the probability of stimulating neighboring fibers which may induce side effects (20). Given differences in individual neuroanatomy, in order to optimize therapeutic effect while minimizing stimulation induced side effects, there is a growing recognition that sculpting the VTA in the x-y plane is as important as defining it in the z-plane. This has led to the development of segmented leads, allowing for directional stimulation (current steering), providing greater precision and selectivity to stimulation regions.

The ability to effectively “steer” the stimulation in specific directions could potentially increase the therapeutic window as well as widen the threshold before inducing side effects (22). In addition, directional leads may also help to optimize the benefit throughout personalized parameters, given that specific “sweet-spot” areas for specific motor and non-motor clinical benefits have been found (23–25). Computational modeling studies have demonstrated that directional leads may have the capability to steer the center of the VTA up to 1.3 mm (22). In addition, when the same amount of current is applied to smaller contacts, a greater charge density is generated. This theoretically would require less overall current to achieve therapeutic benefit and therefore preserve battery life, although the recent development of rechargeable IPG systems has made the need to preserve neurostimulator battery life less relevant (22). Although segmented contacts may have improved spatial selectivity, adjustments must be made to the stimulation parameters to compensate for these changes. Due to the smaller surface area of segmented contacts, the upper limit of stimulation amplitude is lower than a ring contact, so as to avoid current density levels that could cause permanent damage to the surrounding tissue. In addition, since current flows out of the edges of the contact, the use of multiple segmented contacts may reduce the impact of directional stimulation (26). Segmented leads also increase the complexity of programming strategies. One potential solution to improve the efficiency of programming these increasingly complex leads is via automated programming (27, 28). However, further work is needed to determine whether automated programming leads to similar or superior clinical benefit as the traditional DBS programming strategies.

### Implantable Pulse Generators

A typical IPG for DBS weighs between 40–67 g, but there is evidence that these IPGs could potentially be smaller, given that IPG systems for spinal cord stimulators can be as light as 29.1 g (7). A smaller IPG system would not only be more comfortable for patients, especially for those with a smaller body habitus in which the IPG protrudes in the chest or abdomen, but could also potentially lead to the development of cranial IPGs, similar to the responsive neurostimulator (RNS) systems used to treat epilepsy (22). In addition, early IPGs consisted of a single channel device, meaning that one IPG could accommodate one DBS lead, requiring two separate IPGs for bilateral lead implantation (22). More recent IPGs now have dual-lead channel capability, meaning that only one IPG is needed to power bilateral DBS leads.

IPG longevity is dependent on the stimulation parameters used (29). For example, double monopolar stimulation requires significantly more energy than single monopolar stimulation and leads to reduced battery life (30), whereas bipolar stimulation may lead to improved longevity in specific instances (31). Higher frequency, amplitude, and pulse width also correlate with shortened battery life (30). In addition, although designed to deliver a consistent output regardless of their battery status, IPGs reaching the end of battery life (EOBL) often produce lower current outputs than indicated, resulting in a sudden rebound in neurologic or psychiatric symptoms, including potential emergency situations such as dystonic storm in patients with dystonia (7, 32). Unfortunately, models to predict EOBL are not always accurate and an IPG may become fully depleted before replacement, resulting in loss of therapeutic efficacy until the IPG can be surgically exchanged (33).

The development of IPG systems capable of delivering high energy stimulation without depleting the battery life has led to the advent of rechargeable systems. Studies assessing the use of rechargeable IPG systems have found significant improvement in both cost-savings as well as patient satisfaction (33–35). Rechargeable IPG systems are rated to last 15 years before necessitating surgical replacement, leading to an overall reduction in surgical costs (fewer surgical replacements) and device cost (although rechargeable IPGs have a higher unit cost, due to their less frequent need for replacement, the overall cost is less) (33). Drawbacks of rechargeable systems include the need to recharge the unit on a regular basis (several times a week) and occasional technical difficulty with coupling the external charging unit to the IPG. The most recent IPG from Medtronic, the dual channel Percept PC, represents an apparent improvement over the dual channel Activa PC because its casing volume is 20% less with estimated battery longevity averaging over 5 years (36). Several other rechargeable and smaller IPG systems have been developed by other manufacturers (Table 2).

**Table 2:** Overview of IPG systems capable of continuous stimulation, with their features and stimulation parameters.

**Company**	**IPG**	**Rechargeable?**	**Lead Compatibility**	**Frequency (Hz)**	**Pulse Width (μs)**	**MRI Compatibility?**
Medtronic	Activa-PC	N	Dual	2–250	60–450	Conditionally safe
	Activa-RC	Y	Dual	2–250	60–450	Conditionally Safe
	Activa-SC	N	Single	3–250	60–450	Conditionally safe
	Percept	N	Dual	2–250	20–450	Conditionally safe
Abbott	Infinity 5	N	Single	2–240	20–500	Conditionally safe
	Infinity 7	N	Dual	2–240	20–500	Conditionally safe
Boston	Vercise-PC	N	Dual	2–255	10–450	No
	Vercise-RC	Y	Dual	2–255	10–450	No
	Gevia	Y	Dual	2–255	20–450	Conditionally safe
	Genus	N	Dual	2–255	20–450	Conditionally safe
PINS	G102	N	Dual	2–250	30–450	Unknown
	G102R	Y	Dual	2–250	30–450	Unknown
	G101A	N	Single	2–240	30–450	Unknown
SceneRay	1180	N	Dual	1–1600	60–960	Unknown
Newronika	AlphaDBS	Y	Dual	Unknown	<360	Unknown

## DBS Targeting Strategies

A well-positioned DBS lead is instrumental to a successful clinical outcome. While targeting initially relied on finding the appropriate anatomical target, targeting has evolved to focus on the underlying physiologic targetTechniques for surgical targeting vary among institutions, but all involve stereotaxy and image guidance. In addition, many institutions use microelectrode recording (MER) in order to identify the physiologic target. Stereotaxy is used to establish a 3-D coordinate system by which anatomical regions can be precisely and accurately localized and targeted deep within the brain (37). With frame-based systems, a stereotactic head frame is rigidly affixed to the patient's skull after which they undergo a computed tomography (CT) scan to localize the frame and establish a frame-based coordinate system. Commercially-available software can then be used to register (or fuse) a previously obtained volumetric targeting MRI to the head frame CT. This registration establishes a coordinate system by which trajectory planning performed on the pre-operative MRI can be translated to the CT scan with respect to the patient's frame, providing a mechanism for precise, accurate implantation of the DBS lead. Frameless systems employ fiducial markers that are co-registered to pre-operative MRI scans, utilizing a similar mechanism to establish a coordinate system. With imaging advances, the majority of centers have transitioned to using atlas-based direct targeting, where an anatomic atlas is overlaid onto a patient's MRI and linearly or non-linearly deformed to produce a best fit. With the atlas as a reference, a DBS lead trajectory is then planned to maximize the VTA in the target zone. Advances in anatomical atlases, imaging sequences, and connectomics are refining the methods used across centers to improve surgical targeting and to ensure accurate DBS electrode placement for therapeutic benefit.

### Microelectrode Recording

MER has been shown to improve localization of DBS lead placement by using intraoperative recording of electrical activity in different regions of the brain (38). The benefits of MER are clear: MER can safely identify neural structures and borders, MER can help approximate the location within the target that will be most beneficial clinically, and the information gathered from MER can be helpful for understanding disease pathophysiology. MER will likely continue to be an important technique in places that do not yet have access to more advanced imaging techniques (39, 40). Although the use of MER does extend the length of the DBS procedure (41), MER frequently provides important physiologic information that results in lead adjustments up to 20–40% of the time, which can be especially important in instances when there is significant brain shift following preoperative imaging (42, 43).

The accuracy of these adjustments is dependent on the technique used to adjust the DBS lead (40). In addition, as imaging and atlas techniques have improved, the role of MER has been called into question (39). At least one retrospective study has demonstrated no significant differences in mood between asleep and awake DBS cases, (44) but further work with larger, prospective studies are needed to truly compare the benefits of asleep vs. awake cases. Although there is some evidence that advances in interventional MRI technology has led to more accuracy regarding the anatomic placement of the DBS leads, the clinical outcomes between image-guided DBS and MER-guided DBS are similar (45, 46). Further large, randomized clinical studies are needed to determine if and when certain intraoperative lead placement techniques will lead to further clinical benefit. At this time, whether or not image-guided DBS lead placement is superior to MER-guidance remains an important topic for further exploration. From a practical standpoint, it will be important that whichever technique is chosen (image based vs. MER based), appropriate expertise and a quality assurance plan is implemented to ensure the best possible outcomes.

### Anatomical Atlases

Mapping structural anatomical atlases to a patient's anatomy provides a detailed estimate of nuclei borders that may or may not be distinguishable in the imaging. Classical stereotactic atlases, such as the Talairach and Tournoux (47), Schaltenbrand et al. (48), and Schaltenbrand and Bailey (49) atlases, have been digitized and are still commonly employed for DBS targeting. Several new atlases for thalamic and basal ganglia structures have also been more recently developed, including atlases based on histology (50–53), structural or functional connectivity (54, 55), and postmortem or *in vivo* high-field 7T MRI (56–58). Multimodal approaches to atlas construction have also been beneficial for detailed anatomical visualization, as shown in the DISTAL atlas (59). Although the majority of atlases have been developed based on data from healthy controls, population-specific atlases may also provide advantages for capturing specific pathologies, such as the PD25 atlas or the ParkMedAtlas for PD (60, 61). Some atlases also delineate different functional subregions within nuclei; for example, recent atlases identify motor, associative, and limbic subregions of the subthalamic nucleus (62), the globus pallidus internus (63), or the thalamus (64) based on connectivity to their respective networks. Visualizing functional subregions of the target structure adds an additional layer of detail that may be beneficial for DBS targeting.

Atlas selection for DBS targeting depends on several factors, such as the target structure, the indication, the preoperative imaging modalities, and the surgical team preferences. It is important to obtain an accurate registration of the atlas to the patient's brain in order to provide an estimate of the spatial location of the target while accounting for anatomical variability across individuals. Several strategies for improving atlas-to-patient registration have been developed, ranging from manual refinement of fitting (65) to automated algorithms (66). Comprehensive comparisons of different registration techniques have shown that automated non-linear registration algorithms with optimized parameters may yield higher accuracy than other algorithms and also yield similar results to manual segmentations by experts (66, 67). A combination of automated algorithms and manual refinement may be useful for ensuring accuracy. Ultimately, patient-specific factors play a role in determining the most appropriate technique for DBS targeting.

### Imaging Sequences

**Figure 3:**
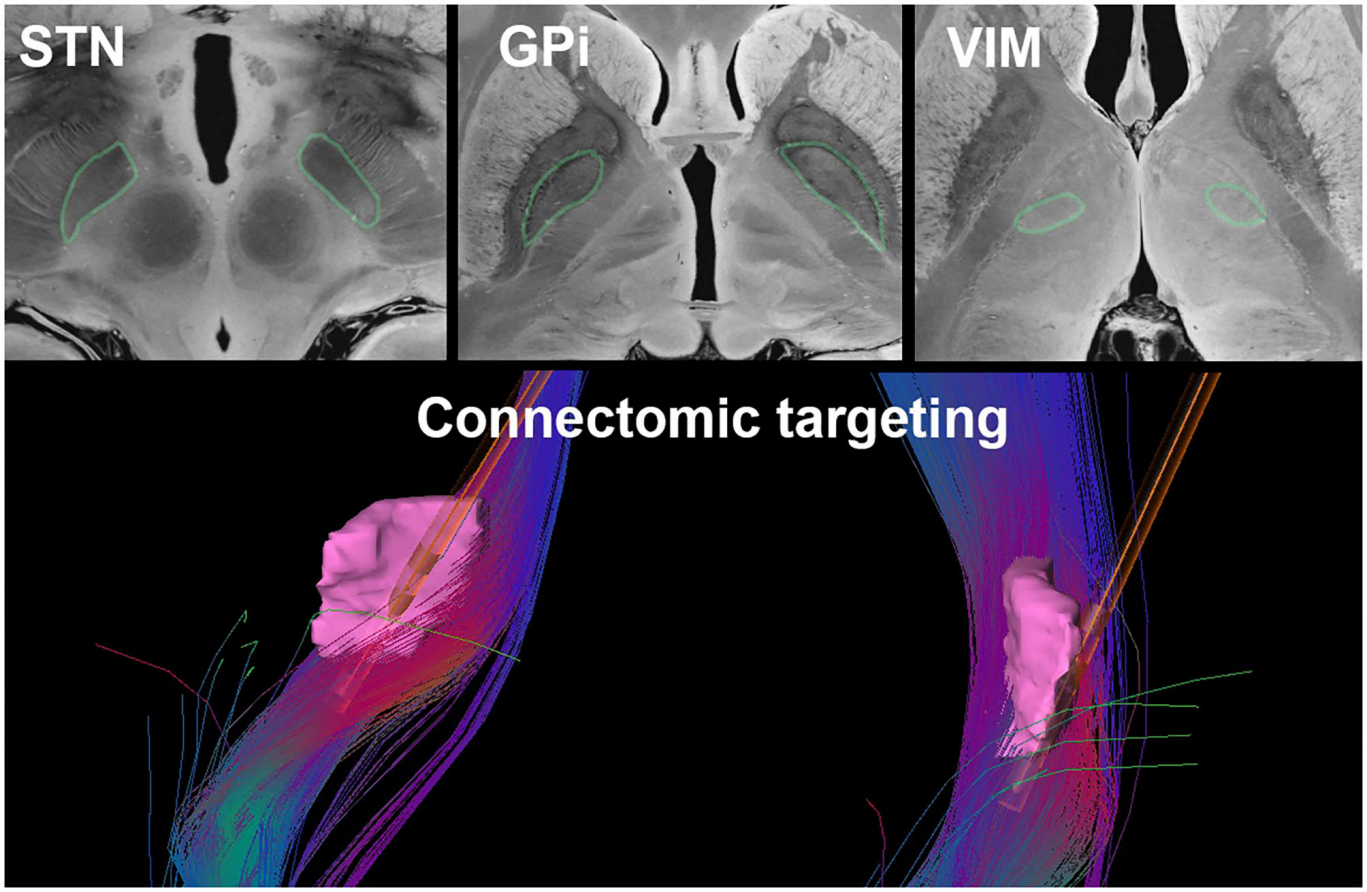
Current DBS targeting strategies such as direct targeting of the STN, GPi, and VIM (outlined in green) as visualized on a 7T MRI of the brain (75) (top) and connectomic targeting of the VIM (pink). The fiber tracts illustrated represent the dentato-rubro-thalamic-tract with a superimposed Medtronic 3387 DBS lead (bottom).

Many novel MRI protocols and processing methods have been developed with the goal of improving visualization of specific anatomical structures. For example, inversion recovery sequences such as the Fast Gray matter Acquisition T1 Inversion Recovery (FGATIR) sequence (68) have been shown to increase contrast in subcortical structures. Quantitative susceptibility mapping (QSM) and susceptibility-weighted imaging (SWI) may also improve direct visualization of basal ganglia structures and thalamic nuclei (69–71). Finally, ultra-high-field imaging with 7T MRI has become increasingly popular due to its higher signal-to-noise ratio, spatial resolution, and structural contrast compared to 1.5T or 3T scanners (72). Ultra-high-field imaging may improve the visualization of the STN (73), the GPi (74), and thalamic nuclei (Figure 3) (58, 76). Currently, 7T MRI scanners are limited in availability to specialized imaging centers; however, as access to these scanners increases, more routine use in neurosurgical planning will become more common.

### Connectomics

In line with our expanding understanding of pathophysiology, the DBS community has moved toward developing “connectomic” neurosurgical targeting approaches (77, 78).

Studies investigating connectomics involve two main components: (1) a model of the effect of stimulation on surrounding neural structures [e.g., the VTA (79, 80) or fiber pathway activation models (81)], and (2) neuroimaging-based connectivity measures to identify brain networks. The model of the effect of stimulation provides an estimate of the spatial extent of activation based on the applied stimulation parameters (contact configuration, amplitude, pulse width, and frequency) and the anatomical location of the DBS electrode. Neuroimaging modalities for deriving connectivity measures most commonly include diffusion tensor imaging (DTI) for structural connectivity and functional MRI (fMRI) for functional connectivity. Combining neural activation models with neuroimaging-based connectivity measures enables direct comparison of the brain networks modulated either across stimulation settings within an individual patient or across a cohort of patients.

Retrospective studies have provided crucial insight into the brain networks involved in symptom improvement with DBS in movement disorders, such as STN or pallidal DBS for PD (82, 83), thalamic DBS for ET (84), and pallidal DBS for dystonia (85). These studies have revealed that connectivity may be used to predict clinical outcomes, supporting the idea that both structural and functional connectivity are important and independent predictors of DBS (82). In addition, these studies have postulated a variety of practical applications of therapeutic connectivity profiles, including determining whether an electrode is appropriately placed and choosing more optimal and patient-specific targets (82, 84). Connectomic analyses of DBS in psychiatric indications has also improved our understanding of the brain networks that may mediate improvement in Tourette syndrome (86, 87), depression (88, 89), and obsessive-compulsive disorder (90). In particular, several studies have shown converging evidence on specific fiber pathways associated with improvement in obsessive-compulsive symptoms across surgical targets (90, 91), and even potentially across Tourette syndrome (87).

Connectomics may also be used to guide DBS targeting prospectively. For example, in DBS for treatment-resistant depression at specialized centers, patient-specific DTI is used to construct fiber pathways in the subcallosal cingulate cortex and the DBS electrode is targeted to the intersection of four critical pathways (forceps minor, uncinate fasciculus, cingulum and fronto-striatal fibers) that have been shown to mediate the anti-depressive response (92). Based on retrospective studies demonstrating clinical benefit in patients whose DBS passed through the aformenetioned fiber bundles, at least one study was able to individualize the connectomic targeting approach in patients with treatment-resistant depression, which could potentially optimize current targeting strategies (92). Another recent study has demonstrated benefits using connectomics to prospectively determine patient-specific striatal DBS targets for obsessive-compulsive disorder based on fMRI with a symptom provocation task (93). Furthermore, for DBS in movement disorders, several prospective targeting methods involving DTI-based connectivity measures have been proposed to refine patient-specific targets (Figure 3) (94–96). Since anatomical targeting can be challenging due to poor landmarks on imaging or unreliable microelectrode recordings, this connectomic approach may offer an alternative or supplemental method of targeting.

Despite numerous retrospective studies investigating brain networks involved in the clinical response to DBS, relatively few have been adopted in clinical practice due to practical limitations. These limitations include increased MRI scan time for specialized sequences, technical expertise necessary for processing the imaging, and specialized software required for integration with established commercial software. In addition, there are still several aspects of this technology which require further refinement before they can be reliably translated into clinical practice. Some of these limitations include distortion inherent to the imaging technique or related to post-processing, motion artifact especially in the setting or patients with movement disorders, and limitations with fiber tracking technology (97). These limitations are especially important to consider given the importance of millimeter-to-submillimeter accuracy in DBS targeting. Given the rapid expansion of connectomic research in DBS, it is likely that connectivity-based targeting will be increasingly used to guide DBS as technology advances and our understanding of the brain networks underlying specific symptoms expands.

## Conventional Stimulation Parameters

There are several parameters used to control the amount of stimulation delivered to the brain that ultimately shape the VTA; the amplitude, the pulse width, and the frequency of the stimulation as well as the polarity of the electrode contact(s) used. These stimulation parameters influence the overall waveform shape contributing to different therapeutic effects.

Conventional stimulation parameters are largely based on structure-effect relationships extrapolated from patients already implanted with DBS (98, 99) and further supported by computational modeling (100). However, the stimulation parameters used in clinical practice often go beyond computational models. The sheer number of combinations of amplitude, pulse width, and frequency can seem overwhelming when preparing to program a patient. With these three settings, the clinicians can create therapeutic waveforms within the recommended charge density limit (30 μC per cm^2^) which was established by previous animal models (101). These numbers have been used as the standard recommendation for decades (102), but recent evidence suggests that they may be too conservative (103). Despite conventional stimulation parameters remaining the mainstay of treatment, individualization of stimulation parameters can in many cases yield further clinical benefit (104).

### Contact Configuration

In addition to these three main stimulation parameters, the polarity of the contacts also plays an important role in shaping the VTA. Current flows between a cathode (-) and an anode (+). Traditional monopolar configurations consist of the IPG assigned as the anode and a single contact assigned as the cathode. Recent technology allows for bipolar and multipolar electrode configuration, which allows stimulation to be shaped with more precision (105). These configurations carry certain advantages. Bipolar or multipolar configurations allow for shaping of the stimulation field with higher stimulation intensities around the cathode(s) and less intense stimulation around the contact acting as the anode, thereby minimizing stimulation in areas that are more prone to side effects.

### Amplitude

The amplitude is often the first parameter adjusted in initial programming visits. A higher amplitude leads to a larger VTA. Therefore, as the amplitude is increased, more fibers are usually affected leading to therapeutic benefit; however, as the VTA increases, surrounding structures may also be stimulated, leading to increased probability of stimulation-induced side effects. Past studies have shown that amplitude is one of the highest correlated stimulation parameters to improvement of motor features of PD in patients with STN-DBS (106, 107). It is often helpful to titrate the amplitude in response to specific symptoms. In PD, rigidity is the most straightforward symptom to monitor because it responds quickly to stimulation and the severity of the rigidity does not fluctuate as much as other symptoms such as tremor (108). In contrast, in order to achieve meaningful clinical benefit for dystonia in GPi-DBS, higher stimulation amplitudes are often necessary compared to STN-DBS for PD (109).

To mitigate the effect of impedance variance of time, newer IPGs have been designed with current-controlled sources, eliminating the influence of impedance on stimulation (22, 110). In this mode, a fixed current source delivers consistent energy to brain tissue irrespective of the system's impedance. A constant-current source in an Abbott IPG was utilized in one of the first randomized controlled trials to evaluate the effect of constant current stimulation in STN DBS for PD (111). This study showed that constant-current stimulation significantly improved quality ON time and motor symptoms at 3 months post-DBS implantation compared to no stimulation (111). The Boston Scientific devices employ multiple independent current control (MICC) technology such that each electrode contact on the DBS lead has its own dedicated power source. This offers increased customizability and flexibility over DBS programming parameters. The MICC technology was evaluated in two large clinical trials in Europe and North America, the VANTAGE and INTREPID studies, respectively (112, 113). In the VANTAGE study, the prospective, non-randomized, open-label study found that UPDRS Part III motor scores improved significantly at 6 months post-DBS implantation compared to baseline (112). In the INTREPID study, the double-blind, randomized, sham-controlled trial found that DBS using MICC technology significantly improved ON time without troublesome dyskinesias at 3 months post-DBS implantation compared to baseline (113). In addition, MICC stimulation has been shown to be clinically beneficial for up to a year (114). Constant-current stimulation is now the preferred option of most experts. At least one small study has directly compared constant current to constant voltage stimulation and found no significant clinical difference between these stimulation techniques (115), and computational modeling studies have also evaluated MICC technology (116, 117). However, larger clinical studies directly comparing MICC to conventional stimulation have not yet been performed.

### Pulse Width

Traditionally, the pulse width of a stimulation pulse is set between 60 and 90 μs (32). More recently, however, shortening the pulse width in patients with STN-DBS for PD (32, 118–120) and for patients with VIM-DBS for ET (121) has been shown to widen the therapeutic window. The authors of one study postulated that a lower pulse width may focus the stimulation on smaller diameter myelinated axons near the electrode as opposed to larger diameter axons located farther away, thus making the stimulation area more precise and potentially also saving battery life (32). In addition, computational modeling has demonstrated that longer pulse widths permit for decreased stimulation amplitudes while maintaining the same neural activation as higher amplitude stimulation trains with pulse widths, ultimately leading to longer battery life (100, 122).

Given the wider therapeutic window at lower pulse widths, there has also been some investigation into whether or not lower pulse widths can also reduce side effects following chronic stimulation. Although technical limitations previously restricted the pulse width to a range of 60–450 μs in early IPG systems, more recent innovations in IPG systems have enabled pulse widths as low as 10 μs. This technical innovation has facilitated continued investigation into how pulse widths can be adjusted to allow for maximal clinical benefit. There was no significant difference in dysarthria when a shortened pulse width of 30 μs was used in patients with STN-DBS (123). However, many patients chose the shorter pulse width option as their preferred setting at the conclusion of the study. A more recent study found no significant difference in motor symptom control on a pulse width of 30 vs. 60 μs for patients with PD and STN-DBS, but patients with dyskinesias preferred the lower pulse width setting (122). Further studies are needed to determine which patient population is most likely to benefit from shorter pulse width programming.

In contrast to STN-DBS for PD, guidelines for programming VIM-DBS for ET are less well-established. Some studies have observed that following optimization of amplitude, longer pulse widths ranging from 90 to 120 μs lead to further tremor suppression (124). However, more recent studies have shown that shortening the pulse width in patients with ET may be a strategy to reduce stimulation-induced side effects such as gait disturbance, ataxia, and paresthesias (125, 126).

Conditions such as dystonia typically require higher pulse widths, sometimes as high as 450 μs, in order to achieve good clinical benefit (109, 124). Although some studies have recommended higher pulse widths in the programming of dystonia (127), other studies have found no significant difference when high or low pulse widths were used for the treatment of generalized dystonia (128). However, since this study only assessed symptoms acutely, it is possible that a higher pulse width may have a more significant clinical impact following chronic stimulation (124).

### Frequency

The rate of stimulation was traditionally delivered at 130 Hz. However, in certain patient populations, adjusting the frequency of stimulation may be an important programming strategy to improve therapeutic benefit or to reduce stimulation-induced side effects. For example, low-frequency stimulation, typically in the range of 60–80 Hz, has been found to reduce freezing of gait and axial rigidity in patients with PD (129–133). However, other studies were unable to replicate these results (134–137). It is unclear if the lower frequency stimulation alleviates freezing of gait or if freezing of gait is a stimulation-induced side effect at higher stimulation frequencies (124). Low-frequency stimulation may also help minimize dysarthria and aspiration risk in patients with PD (138, 139). High-frequency stimulation may be beneficial for tremor-dominant patients with PD who do not already have baseline freezing of gait or significant axial symptoms (137). In addition, there may be a trade-off in improvement of other motor symptoms when lower-frequency stimulation is used (140, 141). Therefore, alternative strategies such as variable frequency stimulation in which high and low frequency stimulation is alternated back and forth, may benefit both freezing of gait as well as other motor symptoms (142, 143). Overall, it is possible that the rate of stimulation will need to be tailored to the specific symptoms with which individual patients manifest. A recent meta-analysis concluded that high-frequency stimulation tends to be better for tremor control and low-frequency stimulation tends to be better for akinesia and freezing of gait in STN-DBS for patients with PD (76). This has not however been adopted widely in clinical practice suggesting that the findings may or may not replicate in the chronic condition. In addition, individual patient responses to different frequencies may not be consistent across patients. Interpatient variability suggests that a wider range of frequencies should be possibly considered in clinical practice (144).

In cases of VIM-DBS for patients with ET, several studies found maximal tremor benefit to be around 100–130 Hz (145–148). Higher frequencies did not significantly reduce tremor amplitude, and rates of >185 Hz were intolerable in some patients (148). In contrast to ET, studies evaluating GPi-DBS for dystonia found that higher frequencies in the range of 180–250 Hz led to significant clinical improvement (149, 150). In patients with dystonia experiencing capsular side effects at the more ventral contacts, however, lower frequency stimulation (80 Hz) may be a programming strategy to improve tolerability of stimulation (151).

### Conventional vs. Novel Stimulation Waveforms

In addition to conventional stimulation parameters, there have been several advances that enable new stimulation approaches, including interleaving, cycling, biphasic, and current fractionation.

Interleaving allows for rapid alternation between two contacts with different amplitudes and pulse widths but the same frequencies (152). This technique can be helpful to avoid stimulation-induced side effects, but this setting can drain the battery at a faster rate. In systems that are FDA approved, the maximum interleaving frequency is half of the maximum non-interleaved frequency. Cycling, in contrast to interleaving, alternates between an active stimulation phase and an off phase, which can also be an effective approach to reducing stimulation-induced side effects. Biphasic stimulation relies on a stimulus pulse phase and an active recovery phase as opposed to a passive recovery phase, which may increase the efficacy of stimulation (153–155). This technique is currently being investigated in various research studies, but is not yet commercially available (156).

Recently, multiple advances have been made in the domain of alternating the temporal feature of the DBS pulse train to achieve better therapeutic outcome or more efficient battery consumption. One of these advances is variable frequency stimulation (VFS) (142, 143). Conventional stimulation may not be as effective for certain symptoms, and using low or high frequencies may be more effective for specific symptoms, so VFS aims to combine the two. Jia and colleagues showed that a combination of multiple frequencies, on the same electrode contact, patterned in blocks can provide better management of both tremor symptoms and axial symptoms (143). VFS paradigms demonstrate that fractional amounts of high frequency stimulation or low frequency stimulation can provide similar benefit to constant stimulation, and may be an important option for certain cases.

Another form of alternative therapy that uses a similar concept but on a much faster and shorter timescale is theta burst stimulation (TBS). TBS has been a common practice in the world of transcranial magnetic stimulation (TMS) (157, 158) and has been shown to provide benefits in PD (159, 160) and dystonia (161, 162). TBS is a stimulation block design that deliver bursts of stimulation that cycles on and off at a rate of 5 Hz. Efforts have been made to bring such therapeutic paradigms to DBS programming (163), but current evidence is preliminary and based on in-clinic observations and may require further testing to address the neuroplasticity effects and long-term observations during chronic stimulation. Additional studies in this area also seek to investigate different burst frequencies for axial symptoms with significantly less battery consumption and fewer side effects (164). However, it is also important to note that not all brain targets benefit from cycling. Swan and colleagues showed that short pauses in thalamic DBS for ET patients promote tremor (165). This means the use of TBS or burst cycling stimulation may require a case-by-case evaluation. On the other hand, temporally optimized patterned stimulation (TOPS) (NCT04390867) is one of the novel therapeutic waveform for DBS in PD patients (166) that is different from previously described VFS. The TOPS algorithm was originally designed to investigate the mechanism of DBS by varying the temporal patterns (interval between pulses) of stimulation. In VIM-DBS for tremor, studies found that a long absence of stimulation leads to worse symptom suppression compared to conventional DBS (167), but a well-organized, temporally irregular stimulation with lower average frequency is able to achieve the same outcome as conventional high-frequency stimulation while reducing the total energy consumed by the IPG (168). These novel waveform paradigms also need to account for wash-in and wash-out periods. For example, some studies have shown that patterns involving cycling bursts, with the frequency maintained at the same level, can lead to worsened clinical outcomes (169). Thus the pattern of stimulation as opposed to simply the frequency itself is an important aspect of programming. Further studies are needed to determine the most appropriate indications for these novel waveforms, as well as to determine whether these stimulation patterns are more efficacious than constant stimulation. The target site itself may also be responsible for differences in wash-out periods, with at least one study demonstrating that therapeutic effects gradually washed out of the zona incerta and abruptly washed out of the STN (170). Thus, there are many variables affecting how the stimulation pattern interacts with the surrounding brain area and the resulting clinical benefit.

## Software Advances

There are several software advances that have improved upon the current clinical programming strategies, including telemedicine, automated programming, and closed-loop DBS.

### Telemedicine: Remote Programming

Telemedicine has been used in the field of medicine over the past few decades (171), but it has only been a recent addition for DBS programming in neurology (172). Remote DBS programming has become especially relevant in the setting of the COVID-19 pandemic when telemedicine technologies underwent rapid expansion to safely deliver healthcare. One of the most important advances that enabled remote DBS programming was the use of better symptom quantification technologies. Such technologies include wearable sensors for objective symptom assessment (173) and advanced video recognition software (174). With objective measurements like these, clinicians are able to gain insight into symptom severity history of the patient prior to the telemedicine visit and to offer suggestions for programming changes before or without the need for video conferencing.

Remote DBS programming has been available through the PINS and SceneRay IPG systems in China since 2017, with a prospective study demonstrating that remote programming of the STN for patients with PD is safe and effective (175). In addition, retrospective analyses have found that not only is remote programming possible, but it saves significant travel time and reduces cost (176–178). Additional studies have focused on the advantages of remote DBS programming during the COVID-19 pandemic, and have found that patients were satisfied with the telemedicine approach and there were no significant adverse events, such as loss of network connections or other software malfunctions (179, 180). During a time when elective procedures often need to be postponed to prioritize patients with COVID-19 and to allocate resources appropriately, telemedicine interrogation of DBS devices may also be an effective way to determine which patients need surgical attention due to issues such as lead migration, software malfunction, or EOBL for the IPG system (181). In 2021, Abbott developed and released the NeuroSphere Virtual Clinic technology, an FDA approved technology for remote DBS programming and communication (26). Although Boston Scientific does not currently offer a remote programming tool to access a patient's individual neurostimulator, the Heart Connect system launched in 2020 allows for a clinician to connect remotely with a DBS expert, share the programming screen, and receive real-time guidance for programming strategies within a patient's local neurology office (182).

The benefits of remote DBS programming are clear: practical advantages for patients such as reductions in travel and cost to clinic visits, improved access for patients in rural locations, enabling frequent DBS programming visits for specific cases that might require frequent titrations, and offers an opportunity to address unintended stimulation side effects with a delayed onset (182–184). However, limitations for this new technology still exist, including difficulty targeting symptoms that are challenging to assess virtually (e.g., rigidity), patient difficulty using technology for remote DBS access, and prevention of potential security breaches of remote DBS platforms (182).

### Automated Deep Brain Stimulation

Automated programming is a new area with the potential to further reduce the burdens and time commitment for both the clinician and patient. The primary focus of automated programming is the use of objective symptom assessment paired with computer-controlled therapy updates (152). One of the common strategies is the use of wearable sensors for tremor (185–187), and more specific tools such as spiral detection for action tremors (188, 189). Recent advancements in sensing technologies has facilitated LFP sensing through embedded neurostimulators to assess continuous changes in biomarkers corresponding to disease states (190). The primary challenge in automated programming, similar to remote programming, is the capability of therapy adjustment in real-time available to the computer running symptom assessment. Most neurostimulators limit which devices are allowed to communicate with it. Research-based devices such as the Medtronic Nexus system is a distributed system with an open Application Programming Interface for amplitude control, and the most recent Medtronic Summit RC+S system offers full programmability for integration with external hardware. However, neither platform are commercially available at this time. A mobile visualization platform has been successfully used by movement disorders clinicians as well as home health nurses with no prior DBS programming experience to successfully choose stimulation settings for patients, with similar outcomes compared to traditional programming strategies (191, 192).

### Closed-Loop Deep Brain Stimulation

Sensing technology is an important and recent update for IPG systems that has the potential to expand closed-loop stimulation to a broad patient population. The Medtronic Percept PC received FDA approval in 2020 and is currently the only DBS IPG system capable of sensing chronic *in vivo* brain activity (36). Local field potentials (LFPs) from deep brain nuclei can be recorded in a natural setting and help understand the underlying neurophysiology of the disease and the mechanisms of deep brain stimulation by identifying physiologic biomarkers of neural dysfunction (36, 193, 194). Further, if LFPs reliably correspond to particular clinical symptoms, then closed-loop technologies can be developed to fine-tune stimulation parameters in real time (26). Identification of pathologic biomarkers upon initial implantation of the lead could also be used to identify contacts likely to yield the greatest benefit when stimulated. Adaptive technology is already used in Europe and Japan and the ADAPT-PD trial in the United States is currently recruiting patients with PD to determine if adaptive DBS technology such as the Percept PC can be safely and effectively used for this purpose (NCT04547712). In parallel to the development of the Percept PC, Newronika developed a rechargeable IPG device with sensing technology called the AlphaDBS system, which recently received CE Mark approval in Europe. A trial is currently underway to evaluate the safety and efficacy of adaptive DBS technology using the AlphaDBS system in 15 patients with PD (NCT04681534).

**Figure 4:**
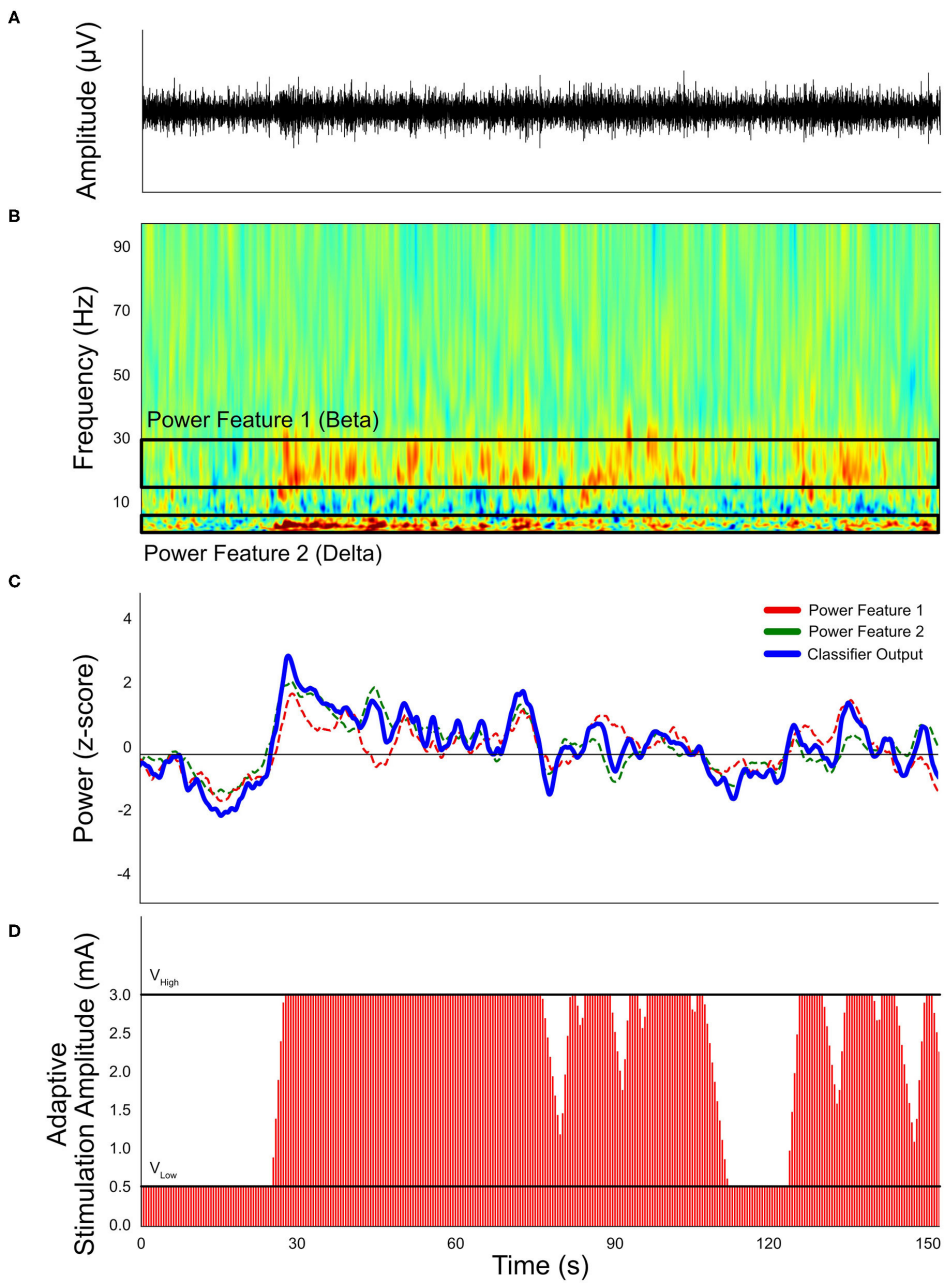
Simulated example of a single-threshold closed-loop stimulation paradigm. **(A)** Neural signals recorded by implanted neural stimulators. **(B)** Transformed spectral content of neural signals. Frequency-band related symptoms are labeled as Feature band 1 (beta) and Feature band 2 (delta). **(C)** Calculated classifier output and proposed threshold. **(D)** Expected stimulation conditions based on the simulated neural signals, based on a 1 mA per second ramp up rate and 0.5 mA per second ramp down rate. The “V_High_” stimulation amplitude is 3.0 mA and “V_Low_” is 0.5 mA.

Sensing technology has led to advances in closed-loop DBS capabilities. Closed-loop DBS can generally be described in two categories: adaptive DBS (aDBS) or responsive DBS (rDBS). aDBS is a form of closed-loop stimulation that adjusts the stimulation amplitude based on the detection of symptomatic events (Figure 4) (195). One of the most common practices is the use of subthalamic beta oscillations as a biomarker for the presence of symptoms (196, 197). For example, beta oscillations have been used as a marker for bradykinesia, with observations that beta power was increased in the off-state and minimized in the on-state in PD. By adjusting the stimulation amplitude based on symptom level, aDBS is able to achieve similar therapeutic outcomes as conventional DBS systems, but with significantly less energy consumption (198) and stimulation-induced side effects (199, 200). rDBS is a common therapeutic strategy for the treatment of epilepsy (201, 202) and has been applied to the treatment of movement disorders (203). The primary difference between aDBS and rDBS is the duration of stimulation after an event is detected: aDBS turns off stimulation when the detector identifies the “disappearance” of the event, but rDBS turns off stimulation after a fixed duration. Although aDBS may offer more precise symptom suppression over rDBS if the symptomatic event lasts longer than pre-defined rDBS duration, rDBS can be used when hardware limitations prevent acute detection of symptom events during stimulation (204).

A variety of biomarkers have been explored. Beta oscillations may be useful as a marker for several reasons, including that beta power correlates with clinical symptoms such as bradykinesia and rigidity, and this correlation is consistent over time (205). Although an exciting field, the use of LFPs as a marker for closed-loop DBS also carries with it several limitations that warrant further exploration in order to improve practicality and accuracy in clinical practice. For example, the LFP signal can be affected by the lesion itself, and sensing the signal at the same time as stimulation can be difficult (206). In addition, beta power may not be representative of all patients. Beta power may not account for all clinical symptoms, and the use of a single oscillatory frequency may be too simplistic to truly capture the physiologic interactions leading to clinical symptoms (207). Further, not all patients manifest with a beta peak, which may lead to inadequate benefit (208, 209).

Stimulation artifacts shadowing the features of event detection are a common problem encountered in LFP-based embedded closed-loop DBS systems. Although there have been many studies focused on eliminating the stimulation artifacts with novel algorithms (210–213), most algorithms are developed in distributed systems with external processors in order to avoid extreme battery drain on the neurostimulators. For embedded algorithms, problem mitigation includes using separate electrodes for feature detection and stimulation (214, 215) to avoid stimulation artifacts, recording brain activity from electrodes equally distant from the stimulation source (“sandwich configuration”), and blanking recording for the duration of the stimulation pulse being delivered (216). The primary consideration of rDBS is the wash-in time (minimum duration of stimulation that offers symptoms alleviation) and typical symptom duration (case-by-case evaluation).

## Looking Toward the Future

The advances in DBS technology have led to exciting implications for the clinical treatment of patients with a variety of disorders. However, it is important to critically assess these technological developments and determine whether these advances also translate into clinical improvements. With the rapid expansion of DBS technology, updates in IPG systems have led to increased flexibility with programming strategies and rechargeable capabilities. These advances in combination with segmented leads offer an overwhelming potential of stimulation paradigms. In addition, closed-loop forms of stimulation are on the horizon with reports of beneficial clinical outcomes. However, more extensive work is needed to determine which patients will benefit most from these types of technology, and a systematic approach to programming is needed in order to more efficiently determine optimal stimulation parameters. Direct comparisons of targeting strategies, including between awake and asleep DBS, as well as between different imaging strategies are needed. These comparisons should include both short-term and long-term follow-up so that clinical benefit can be assessed at multiple timepoints. In addition, risk of utilizing any of these new technologies should be formally assessed and weighed against potential benefit. In an increasingly connected world, it will also be important to preserve the security and privacy of patients who have technology capable of remote programming. This is especially relevant in the current pandemic, when telemedicine has become an increasingly important way of communicating in the healthcare world.

Further larger and prospective studies are needed to evaluate many of these technological advances in further detail. Implementation of patient registries, modeled off of the International TS DBS Registry, may be one solution to help answer these questions, especially given institutional variability that may be based on access to resources or expertise. The goal of an international patient registry would be to allow for data sharing across multiple centers, enable practitioners to more readily share recommendations regarding stimulation paradigms and targeting strategies, and engage in more standardized, multi-center studies on a larger scale. In addition, Big Data analysis and Artificial Intelligence are strategies that may help to reveal patterns across large amounts of data regarding various DBS technologies.

## Conclusion

DBS has undergone an extensive and rapid evolution. Advances in atlases, imaging techniques, and connectomics have collectively improved DBS targeting strategies. Improvements in lead design has allowed segmented contacts to be used for directional stimulation, and improvements in IPG design have led to smaller, longer-lasting, batteries that are MRI compatible. Advances in software have enabled a variety of programming strategies to be employed to help improve efficacy while minimizing stimulation-induced side effects and also by maximizing battery life. Looking toward the future, brain sensing will help clinicians and researchers understand the physiologic aspects of DBS and potentially act as another programming strategy. Closed-loop DBS may help to tailor stimulation parameters to individual symptoms. Additionally, remote and virtual programming may become a more feasible and accessible option. DBS technology is now applied broadly to a wide range of diseases and symptoms, and research is underway to improve upon current designs.

## Author Contributions

JF: contributed to the writing of the first draft, conceptual organization, literature review, and major revisions. JC, KJ, and JW: contributed to writing of the first draft, revisions, and figure illustration. JH, CB, and MO: contributed to revisions. CH: contributed to major revisions and conceptual organization. All authors contributed to the article and approved the submitted version.

## Funding

JF reports grants from the Dystonia Medical Research Foundation, unrelated to this article. JW's research was supported by NIH R25NS108939.

## Conflict of Interest

The authors declare that the research was conducted in the absence of any commercial or financial relationships that could be construed as a potential conflict of interest.

## Publisher's Note

All claims expressed in this article are solely those of the authors and do not necessarily represent those of their affiliated organizations, or those of the publisher, the editors and the reviewers. Any product that may be evaluated in this article, or claim that may be made by its manufacturer, is not guaranteed or endorsed by the publisher.
